# Initial health assessments of children and young people seeking asylum and refugees in Europe: insights from a qualitative study of health care providers

**DOI:** 10.1007/s00431-025-06431-y

**Published:** 2025-09-12

**Authors:** Marta Alustiza, Sophie Pach, Shunmay Yeung, Nicky Longley, Sarah Eisen

**Affiliations:** 1 Division of Paediatric Infectious Diseases, Dr. von Hauner Children’s Hospital, LMU Klinikum, Munich, Germany; 2 Department of Clinical Research, London School of Hygiene & Tropical Medicine, London, UK; 3 Whittington Health NHS Trust, London, UK; 4 Hospital for Tropical Diseases and Children and Young People’s Division, University College London Hospitals NHS Foundation Trust, London, UK

**Keywords:** Refugee, Asylum-seeking children, Unaccompanied minor, Immunisation, Europe, Tuberculosis

## Abstract

In 2023, the number of people seeking asylum globally reached a record high, with 38% aged under 18. Over the past three decades, Europe has experienced the largest increase in international migration since records began. However, research focused on the health of migrant children remains limited. While European guidelines for their care exist, evidence regarding the implementation of these recommendations is limited. This study explores practices and experiences of health care professionals in Europe who conduct initial health assessments (IHAs) for children and young people seeking asylum and refugees (CYPSAR). A qualitative study was conducted (between July and August 2022), using questionnaires and semi-structured interviews to explore perspectives of health care providers WHO care for migrant children in Europe. Participants were recruited through snowball sampling from European expert networks, with 16 clinicians from eight European countries taking part. Data were analysed thematically using NVivo12. We found considerable variation in the content and delivery of IHAs, particularly with regard to screening for communicable and non-communicable diseases. Although immunisation was prioritised, some services lacked vaccination capabilities. Mental health services were limited, with minimal standardised screening. Key barriers to care included insufficient documentation, limited funding, staffing, patient relocation, and inadequate infrastructure to support migrant health. Access to professional translators and the involvement of multidisciplinary teams were considered the main facilitators.

*Conclusion*: Care delivery for migrant children across Europe remains variable despite existing guidelines. Significant barriers to delivery of equitable, quality care exist. There is a need for resources tailored to address challenges in migrant health. Further research is needed to inform evidence-based practice and achieve high-quality equitable care for migrant children in Europe.

**Table:** 

**What is Known:** • *Children seeking asylum and refugees face particular health challenges shaped by experiences before, during and after migration.* • *Recommendations for initial health assessments upon arrival in European host countries exist, but their implementation across different settings remains variable*.
**What is New:** • *While the content and delivery of initial health assessments for children and young people seeking asylum and refugees vary across Europe, clinicians consistently identify common barriers and facilitators to health care.* • *Our findings emphasise the need for an evidence-based approach to support effective, consistent delivery of services to this vulnerable group and provide insight into factors which may influence this.*

**Supplementary Information:**

The online version contains supplementary material available at 10.1007/s00431-025-06431-y.

## Introduction

In 2023, there were 36.4 million refugees worldwide, and 6.1 million people sought asylum—the highest number on record [1, 2]. Of these, 38% were under 18 years (children and young people seeking asylum and refugees; CYPSAR) [2]. The rise in international migration to Europe has been more significant than in any other region over the past three decades [2]. In 2022, 19% of people seeking asylum in Europe were unaccompanied minors (CYPSAR-U), often referred to as unaccompanied asylum-seeking children (UASC) [3]. Ongoing geopolitical instability, including conflicts in Sudan, Ukraine, and the Middle East, indicates that child migration to Europe is likely to continue increasing.

Under the Convention on the Rights of the Child (CRC), signed by all European countries, children seeking asylum or in an irregular situation are entitled to the same rights as those with legal residency. The CRC specifically affirms the child’s right to enjoy the highest attainable standard of health and to have access to treatment and rehabilitation for illness [4].

Most CYPSAR arrive in Europe after long, arduous journeys with limited access to care [5] and present with health needs that may differ from those of children born in Europe [6–8]. Therefore, the WHO recommends a holistic and comprehensive health assessment delivered by a health care worker with a background in paediatrics as soon as possible after arrival to the destination country [5].

Although evidence related to, and research into, migrant child health remain scarce, several European recommendations for the care of CYPSAR exist, including the consensus recommendations of the European Academy of Paediatrics (EAP), based on the recommendations of 31 European countries [9]. However, research on practice and implementation of existing recommendations across Europe is lacking. This study aims to explore how IHAs for CYPSAR are conducted across Europe, specifically examining their content, and perceived barriers and facilitators to quality care delivery, using qualitative methodology to explore experiences of clinicians across diverse European settings.

## Methods

This is a qualitative research study, in which data were collected using a questionnaire and semi-structured interviews following the Consolidated Criteria for Reporting Qualitative Research (COREQ) guidelines [10].

The literature on migrant children uses a variety of terms (asylum-seeker, refugee, displaced) interchangeably. Here, we use the term CYPSAR to mean a child or young person (< 18 years) who has moved to or settled in another country as a result of unfavourable conditions, including war and violence, socioeconomic deprivation, health care, or education limitations [9, 11].

### Study population and sampling

The target population was clinicians working in European (European Union member states and UK and Switzerland) countries who provide IHA for CYPSAR. Eligible participants were initially recruited through recognised European networks of clinicians with expertise and experience working with this population (EuroTravNet, European Society of Paediatric Infectious Diseases), national paediatric associations, and snowball sampling. Potential participants were invited to consent to participate by email. Once recruited, a questionnaire was emailed, and an interview date scheduled.

A sample of 15 participants was deemed sufficient to achieve thematic coverage appropriate to the study’s exploratory aims [11]; however, participants who responded beyond the point of active recruitment were still allowed to participate.

### Data collection

Data were collected between July and August 2022. Structured questionnaires and a topic guide for semi-structured interviews were designed by the investigators based on European recommendations for health assessment of migrant children [9]. Questionnaire responses were collected via Google Forms, then pseudo-anonymised using a participant code. Interviews were performed online via Zoom (version: 5.7.6) and transcribed (Happy Scribe audio transcription platform).

The principal researcher (MA) pilot-tested the questionnaire and interview guide with a UK doctor with experience in refugee health care to refine prior to use.

#### Questionnaire and semi-structured interview procedure

A questionnaire in English, distributed by email, was used to collect specific factual descriptive data about participants and the services in which they worked (Appendix 1). Responses were then discussed in-depth in interviews conducted by MA. No prior relationship had been established between the participants and MA, and participants were informed about MA’s background and interest in the study. No non-participants were present during interviews. Interviews were conducted using semi-structured, open-ended questions based on a topic guide (Appendix 2). Interviewees were encouraged to introduce new topics and themes they deemed relevant. Interviews were video-recorded, lasting 50–90 min. No repeat interviews were conducted, field notes were not made during interviews, and transcripts were not returned to participants.

### Data analysis

Questionnaire data were summarised and triangulated with themes identified in interviews. Transcripts were proofread against recording and uploaded to NVivo12 (QSR International Pty Ltd 2018). Content thematic analysis was used (by MA) to analyse data and themes [12].

After familiarising with the data, three interview transcripts were analysed using both deductive and inductive (open) coding to develop an initial coding framework [13]. Codes were grouped into themes informed by the literature and dataset, then refined into a final framework (Appendix 3) and applied to all data. Key themes were summarised for reporting. Participants did not provide feedback on the findings.

### Ethical consideration

Ethical approval was obtained from the London School of Hygiene and Tropical Medicine (ref. 27582). Informed consent was provided by all study participants. Study records were securely stored with restricted access, and no participant identifiable data were shared.

## Results

### Participant demographics

The study included 16 participants from eight European countries, working in different specialties and settings (Table 1). Half were specialist medical doctors in Paediatric Infectious Disease; the remainder represented a range of other specialities.

**Table 1:** Demographics and professional backgrounds of study participants

ID Country Speciality Level of care			
Informant 1 (I01)	Switzerland	Paediatric Infectious Diseases	Tertiary
Informant 2 (I02)	Spain	Paediatric Infectious Diseases	Tertiary
Informant 3 (I03)	UK	Paediatric Infectious Diseases	Secondary
Informant 4 (I04)	UK	Community Paediatrics	Other^1^
Informant 5 (I05)	Netherlands	Global Paediatrics	Tertiary
Informant 6 (I06)	Austria	Paediatric Infectious Diseases	Tertiary
Informant 7 (I07)	Germany	General Paediatrics	Primary care
Informant 8 (I08)	Germany	Paediatric Infectious Diseases	Tertiary
Informant 9 (I09)	Germany	Tropical Medicine	Tertiary
Informant 10 (I10)	Germany	General Paediatrics	Other^1^
Informant 11 (I11)	France	Infectious Diseases	Tertiary
Informant 12 (I12)	France	Paediatric Infectious Diseases	Tertiary
Informant 13 (I13)	Netherlands	Youth Health Care	Other^1^
Informant 14 (I14)	Spain	Paediatric Infectious Diseases	Tertiary
Informant 15 (I15)	France	Paediatric Infectious Diseases	Tertiary
Informant 16 (I16)	Greece	General Paediatrics	Tertiary

^1^These services were located outside the standard health system framework (i.e. refuge shelter or preventive medicine)

### Key themes

Key themes identified are summarised in Table 2. These are explored below, first in terms of description of the service provided and then with regard to barriers and facilitators to providing IHAs.

**Table 2:** Key themes

Key themes	Subthemes
1. Service structure	Setting. Level of care provided by and expertise in the service
	Health care pathway and clinical presentation/route of referral
	Patient characteristics
	Access, coverage, and funding
2. Features of the health assessment	Medical history and physical examination
	Immunisations
	Physical health (screening for infectious diseases)
	Physical health (non-communicable diseases)
	Mental health
	Sexual health
	Assessment duration
3. Resources within the service	Staff
	Translation services
	Documentation
	Guidelines and training

### Description of the service

#### Service structure

**Fig. 1:**
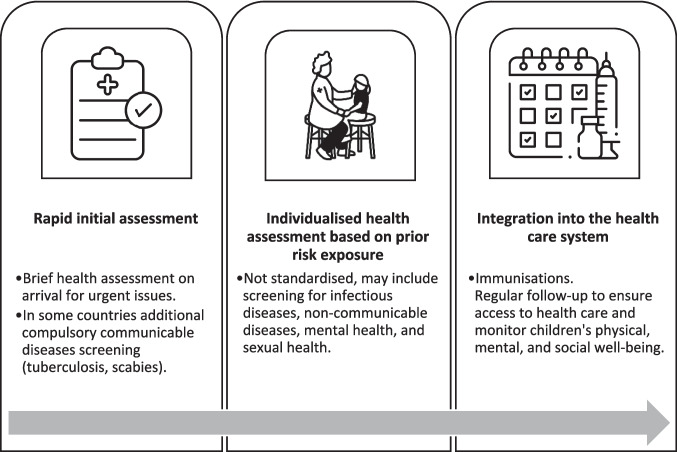
Health care assessment stages for CYPSAR after arrival in European host countries^1^

Participants provided health assessments of CYPSAR in a variety of settings, including refugee shelters, hospitals, and community-based services, and encountered CYPSAR at different points in the patient journey. Encounter types are summarised in Fig. 1. Some performed rapid standardised post-arrival assessments, usually with a focus on infection screening; others a holistic IHA (often in a hospital setting), with onward referral to primary care for immunisations and follow-up.

#### Demographics of service users

Multiple countries of origin of assessed CYPSAR were described, most commonly Afghanistan and Syria. All informants anecdotally reported encountering more male than female patients. Some described their services as only or predominantly providing care to *either* CYPSAR who are unaccompanied or to families with young children, others worked with both groups.

#### Features of initial health assessment

Most IHAs included a medical history and physical examination. Immunisation was offered in some settings, others referred to primary care or other services to complete immunisations (Table 3).

**Table 3:** Main features of initial health assessments

	**History taking**	**ID-screening**	**Possibility to immunise within the same service**	**General health blood screening (including full blood count, haemoglobinopathy, vitamin D)**	**Mental health screening**	**Duration**^**†**^
I01	Doctor	Yes	Always	In all patients	HEADS^1^	60 to 90 min
I02	Nurse/doctor	Yes	Always	In all patients	Unstructured	Nurse 20 min Doctor 20 min
I03	Doctor	No	Always	In all patients	SDQ ^2^	60 min
I04	Doctor	No	Always	In all patients	RGS3	90 min
I05	N/A ^5^	No	Occasionally	In symptomatic patients	CRIES^4^	N/A ^5^
I06	Doctor	No	Always	In all patients	Unstructured	60 min
I07	Doctor	Yes	Occasionally	Based on country of origin	Unstructured	20 min
I08	Doctor	Yes	Often	Rarely	Unstructured	30 min
I09	Questionnaire/doctor	No	Always	In all patients	Unapproached	15–20 min
I10	Doctor	Yes	Always	Rarely	Unstructured	45 min
I11	Doctor	Yes	Always	In symptomatic patients	Unstructured	N/A ^5^
I12	Doctor	No	Always	In all patients	Unstructured	45–60 min
I13	Doctor	Yes	Never	Rarely	Unstructured and SDQ^2^	60 min
I14	Doctor	Yes	Always	In all patients	Unstructured	30 min
I15	Doctor	Yes	Always	In all patients	Unstructured	35 min
I16	Doctor	Yes	Sometimes	In all patients	Unstructured	45–60 min

*ID* infectious diseases, *NCD* non-communicable diseases

*Mental Health Screening Tools*: ^1^HEADS-Questionnaire ([14]); ^2^The Strengths and Difficulties Questionnaire (SDQ) ([15]); ^3^RGS 15-Refugee Health Screener 15([16]); ^4^The Children’s Impact of Event Scale - CRIES-13 ([17]); ^5^N/A, not applicable

^†^Duration of the first consultation

Both infectious diseases screened for and context for screening varied significantly, as shown in Table 4. Some participants offered universal asymptomatic screening, whilst others screened based on symptoms or country of origin. Tests for HIV, tuberculosis (TB), hepatitis B, and hepatitis C were frequently offered universally. In many cases, TB screening was carried out by independent services, most commonly with a chest radiograph for those over 15 years and a tuberculin skin test or interferon gamma release assay test in younger children. However, there was significant variation in practice.

**Table 4:**
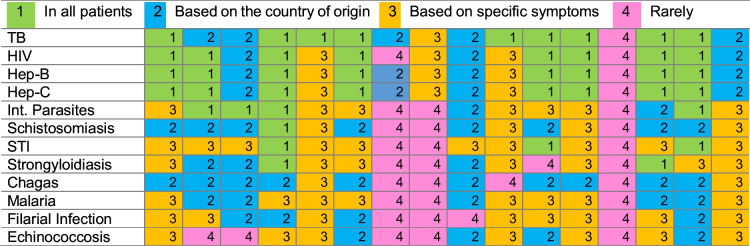
Details of the infectious diseases screening protocol described by participants

*TB* Tuberculosis, *STI* Sexually transmitted infections, *HIV* Human immundeficiency virus

Testing for intestinal parasites and neglected tropical diseases such as schistosomiasis and strongyloidiasis was usually based on symptoms or country of origin. Three services routinely treated patients empirically with anti-helminthics.

Of 16 participants, 13 reported conducting a developmental assessment, particularly those who commonly saw families with young children. Many offered nutrition advice, whilst only three participants mentioned performing vision or hearing tests. With regards to routinely performed investigations (screening for anaemia, haemoglobinopathy, and vitamin D levels), 11 services routinely tested haemoglobin for anemia, whilst six tested vitamin D.

Almost all participants discussed mental health and trauma during the IHA. Most took an unstructured approach through questions regarding quality of sleep, headaches, or school performance. However, some interviewees used specific questionnaires (Table 3) to screen for mental health problems.

Only three services offered universal testing for sexually transmitted infections, ten offered symptomatic screening only, and the three remaining sites did not routinely offer testing (Table 4). Sexual health discussions, addressing infection, safety, consent, and abuse, were offered in most, but not all, services seeing adolescents. Only a few services directed patients to sexual health services. Some informants discussed female genital mutilation (FGM), although most did not.

#### Resources within the service

Health reports were provided in the national language across all services. In some cases, comprehensive reports were sent to patients after follow-up consultations, while in others, brief reports were given directly to patients. However, not all services provided written documentation to service users. All but one respondent had access to translation services, including face-to-face, telephone, and video-call options. Participants reported significant variation in staffing models (Table 5).

**Table 5:** Summary of barriers and facilitators to the successful delivery of health assessments for CYPSAR by theme

Theme	Subtheme	Barriers	Facilitators	Quotations
1. Service structure	Level of care	Lack of resources in primary care and challenging follow-up if hospital-based	Proximity to community, better follow-up and network between primary and tertiary care	*“They were getting lost in the system.”* *”Our first visits are 60 to 90 min (…) which is impossible in primary care.”*
	Access	Language barriers, system complexity, bureaucracy	Migrant-focused services	
	Coverage	Delayed coverage	Free services regardless of status	
	Funding	Underfunded services, lack of data for advocacy	Specific schemes, data collection networks	
2. Features of health assessment	Immunizations	No access to vaccination history, serology challenges	Empirical vaccination, access to origin-country schedules	*"We want that (perform the ID Screening), but it is about money (…) and it is very political."* *"If someone tells us that they have been in prison (…) you assume they have been abused."* *“We have spent a lot of time locating them in camps.”*
	Infectious diseases	Screening infrastructure and coordination gaps	*None reported*	
	NCDs^1^	Language barrier, loss to follow-up despite need to care	*None reported*	
	Sexual health	Cultural taboos, privacy and trust	Referral to specialised clinics with expertise in FGM^2^	
	Mental health	Lack of trained staff, trauma unaddressed	Civil society, early school enrolment, access to psychologists or trained nurses	
	Assessment duration	Time intensive	Nurse-led, questionnaire-supported intake	
3. Resources in the service	Documentation	Missing or unclear past records	Migrant-specific health booklet	*“For us, it [the guideline] is very important; otherwise, we can never make our case with the government.”* *” Sometimes I feel overwhelmed. This is one of the most difficult things I do as a doctor.”*
	Translation	Limited access, restricted languages, scheduling	Culturally aware interpreters, telephonic anonymity	
	Staff	Shortages, over-reliance on doctors	CHWs^3^, trained nurses, case coordinators	
	Guidelines and training	Non-adapted to resources, limited access to trainings	National guidelines, expert networks, background in ID^4^ and tropical medicine	
	Teamwork and networks	Emotional burden, lack of communication between health sectors	Professional societies, team support	
4. Other factors	Living conditions	Homelessness, poor living conditions	Civil society support	*“Recently, I had to release a child, I think, one year old, in the street.”*

### Barriers and facilitators

Participants described a range of barriers and facilitators encountered during IHAs. Table 5 summarises these by theme and includes selected key quotations. A more detailed table with additional quotations is provided in the supplementary materials (Appendix 5).

## Discussion

We present qualitative data from 16 clinicians working with CYPSAR in a range of settings in eight European countries. There was significant variation in the content of IHAs offered to migrant children, with regard to assessment for both infectious and non-communicable diseases, including mental health. Interviews revealed significant shared experience regarding barriers and facilitators to delivery of high-quality IHAs despite variation in settings and available health care provision.

Similarities in services provided by our participants include a common approach of history and examination, as well as inclusion of immunisation history in the health care assessment. This overall structure is in line with European [9, 18] and international recommendations [5, 11]. However, infectious diseases screening offered varied widely, and many settings only offered TB screening universally. This is consistent with recent surveys of infection screening in European migrant populations [19–22]; however, there is evidence to suggest that screening for a wider panel of infections may be more effective [21, 23], particularly in the CYPSAR-U population [24, 25]. Evidence suggests significant inter-country [26], as well as intra-country, variation in recommendations and practice for infection screening (for example, regional variation within Germany) [27].

Nearly all participants performed some form of mental health assessment. Approaches varied widely from unstructured to structured. However, most informants reported significant resource challenges in access to adequate mental health care to address issues identified at assessment. This is consistent with existing literature, which shows that mental health problems are very common in this population [28–31] as a result of traumatic experiences in both countries of origin and transit [32] and that, currently, mental health provision for migrants is under-resourced in most European countries [22, 33–35]. Participants supported the use of a mental health questionnaire, which is consistent with recent research [16, 36] and guidance [11, 37]. Further research is needed into early effective and cost-effective mental health stabilisation in this vulnerable and traumatised population.

Despite wide variation in services offered, experiences of barriers and facilitators to high-quality IHAs were largely consistent amongst participants. Informants emphasised that extended and in-depth consultations were often necessary due to the need for interpreters, complex patient histories, and exploration of unstable social situations. As a result, challenges around resources and funding were nearly universal. Furthermore, most interviewees reported inadequacy of documentation systems posing challenges for effective cross-sectoral information sharing.

Facilitators to providing complex consultations were also consistent with those described elsewhere. Participants emphasised the importance of qualified professional translators, consistent with current understanding of factors addressing cultural barriers in migrant health care [38, 39]. The value of multidisciplinary teams, including nurses, community health workers, and safeguarding and social care teams in facilitating appropriate IHAs was also highlighted, in line with recent literature promoting a ‘whole child’ approach to IHAs for migrant children [6, 40]. Based on the literature, it is also important to listen to the voices of service users in service development [39, 41, 42]. Further research is needed into cost-effectiveness of various models of care and the use of peers and lower cost staff to support delivery of IHAs.

Our findings highlight variability in health assessments across Europe and lack of standardisation in practice, which is not explained by differences in national guidelines, which are very similar in their content [19, 43–46].

This variability may result from modifiable factors, including resource limitations, lack of evidence regarding specific needs of migrant children [20], challenges in implementing existing guidelines—often due to political context and funding [23], and inter-country differences in national policies and practices [22]. Non-modifiable factors may include varying demographics and health profiles of arriving children, which influence service needs and priorities [20, 22, 23, 47].

### Limitations

While our analysis, conducted across 16 interviews, indicated that theoretical sufficiency was achieved—as new data ceased to yield novel themes or significant conceptual insights relevant to our research question—we acknowledge that the current manuscript does not provide a detailed account of the qualitative indicators used to establish this. A more explicit articulation of this iterative process would have further strengthened our claim regarding data adequacy, aligning with recommendations for transparency in thematic analysis [47]. However, an important limitation of the study is possible reflexivity, as all coding and interviews were performed by the same researcher, who, at the time of the study, was a MSc student with a background in paediatrics. Further, not all European countries were included, and some (e.g. Germany with four participants) were represented more than others (e.g. Switzerland and Greece with one participant each). Most of the included countries were from Western Europe, which may further limit the generalisability of our findings, within Europe and beyond.

## Conclusions

We show that IHAs for migrant children are delivered in a range of settings and services across European countries. Despite some commonality in content and approach, there is wide variation in implementation. There is significant shared experience, however, around factors which may make delivery of care challenging and factors which mitigate against these.

There is a need for further standardised data collection to determine and evidence best practice and inform robust guidelines to ensure equitable and consistent delivery of care to this vulnerable population. It is imperative that the voice of the child should be central to this approach.

Evidence, advocacy, and resource are needed for the development of appropriate, high-quality services for CYPSAR, which should include the following key components:

1. Multidisciplinary teams with the time and expertise to address complex and intersecting physical health needs (including infectious diseases screening and immunisations) and to identify mental health, sexual health, and safeguarding concerns, as well as address broader social determinants of health in this cohort.
2. Access to trained interpreters for both language and cultural mediation, alongside access to specialised mental health and sexual health services.
3. Clear and consistent cross-sectoral communication, both within and outside the host country, to promote continuity of care.
4. Support for integration into the regular health care system, particularly through linkage with primary care services, to ensure long-term follow-up and equitable access to health care.

It remains a significant challenge to adequately support this group within standard health care services in most European countries. There is a need for investment in migrant child health services to ensure an adapted quality health assessment but also to support the integration of these children into standard health care systems for ongoing care following initial health assessment.

## Supplementary Information

Below is the link to the electronic supplementary material.

Supplementary Material 1 (DOCX 1.11 MB)

## Data Availability

No datasets were generated or analysed during the current study.

## Abbreviations

CYPSAR: Children and young people seeking asylum and refugees
CYPSAR-U: Children and young people seeking asylum and refugees-unaccompanied
CRC: Convention on the Rights of the Child
EAP: European Academy of Paediatrics
FGM: Female genital mutilation
HIV: Human immunodeficiency virus
Hep B: Hepatitis B
Hep C: Hepatitis C
ID: Infectious diseases
IGRA: Interferon-gamma release assay
IHA: Initial Health Assessments
NCD: Non-communicable diseases
PSA: People seeking asylum
STI: Sexually transmitted infections
TB: Tuberculosis
UASC: Unaccompanied asylum seeking children
WHO: World Health Organization
