# Supplementary material for: Initial health assessments of children and young people seeking asylum and refugees in Europe: insights from a qualitative study of health care providers
Source: Eur J Pediatr. 2025 Sep 12;184(10):612. doi: 10.1007/s00431-025-06431-y (PMC12426126; doi:10.1007/s00431-025-06431-y)
Supplement: Supplementary file 1 — Supplementary Material 1 (DOCX 1.11 MB) [file 431_2025_6431_MOESM1_ESM.docx]

**Supplementary information**

**Appendix 1: Questionnaire**

**
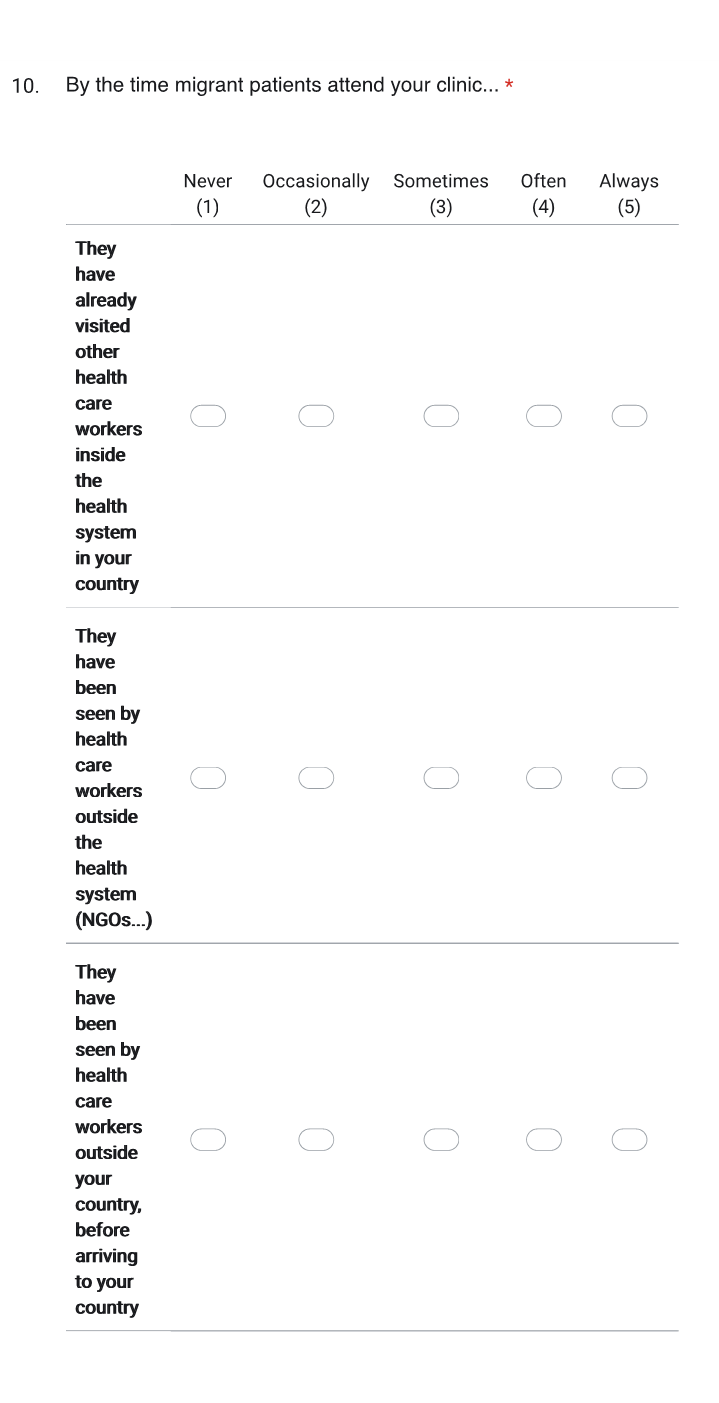
**

**
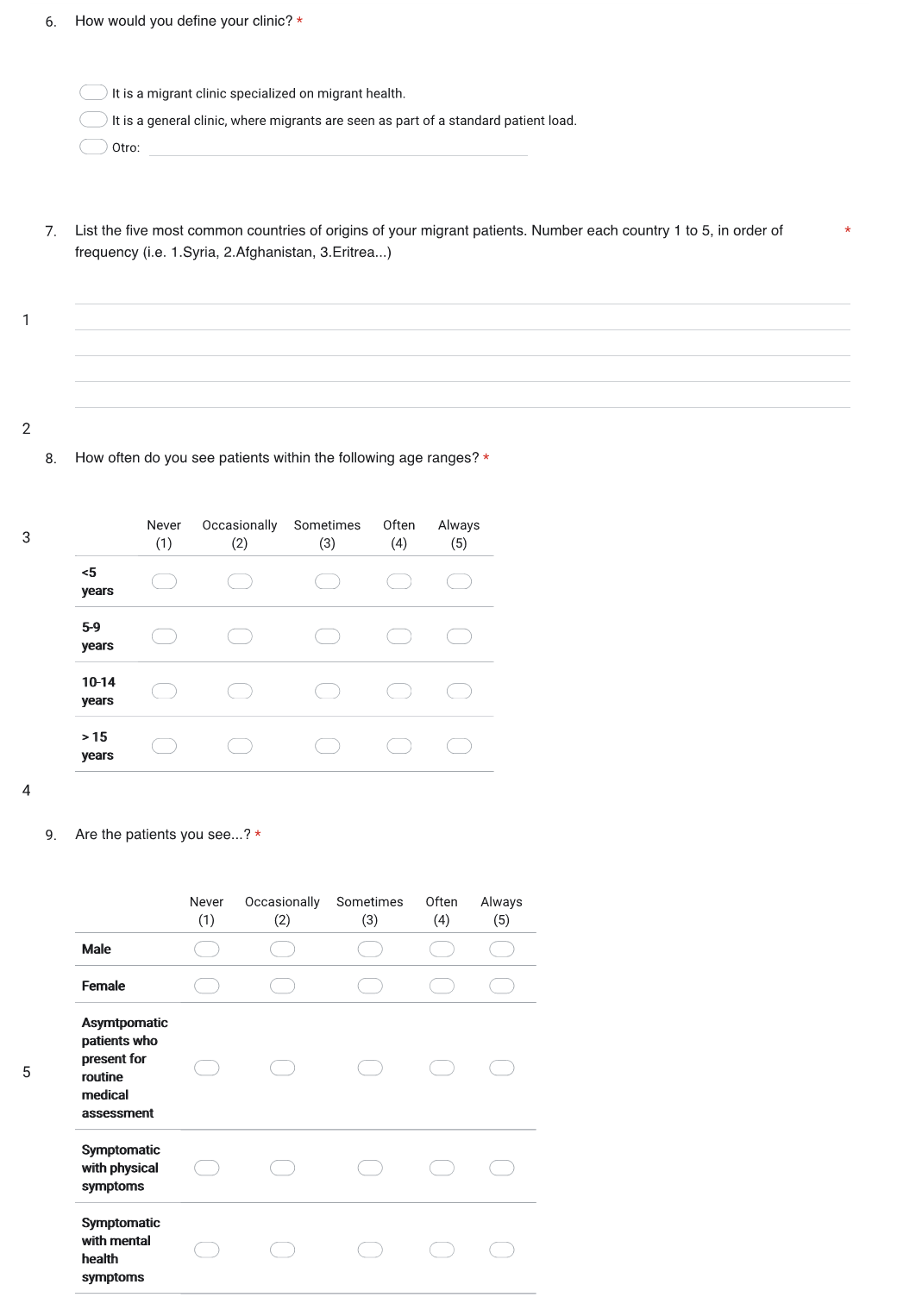
**

**
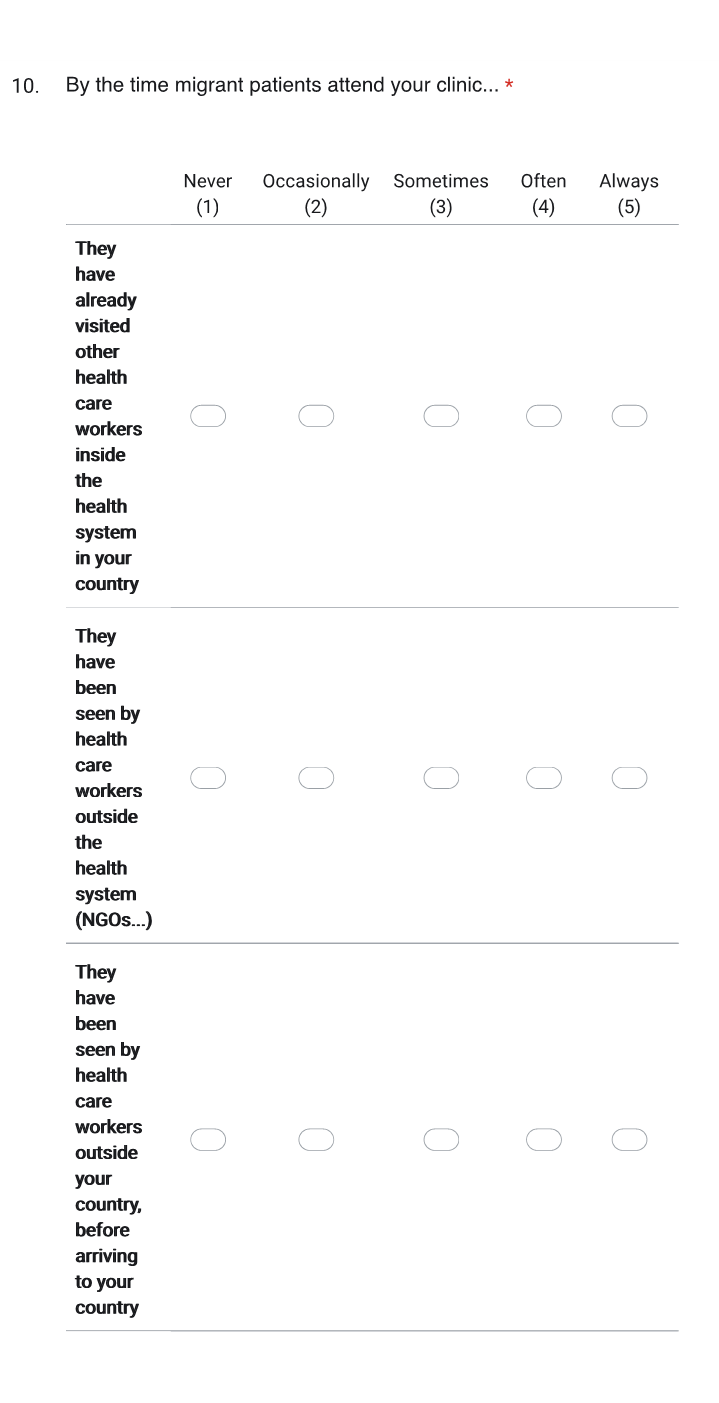
**

**
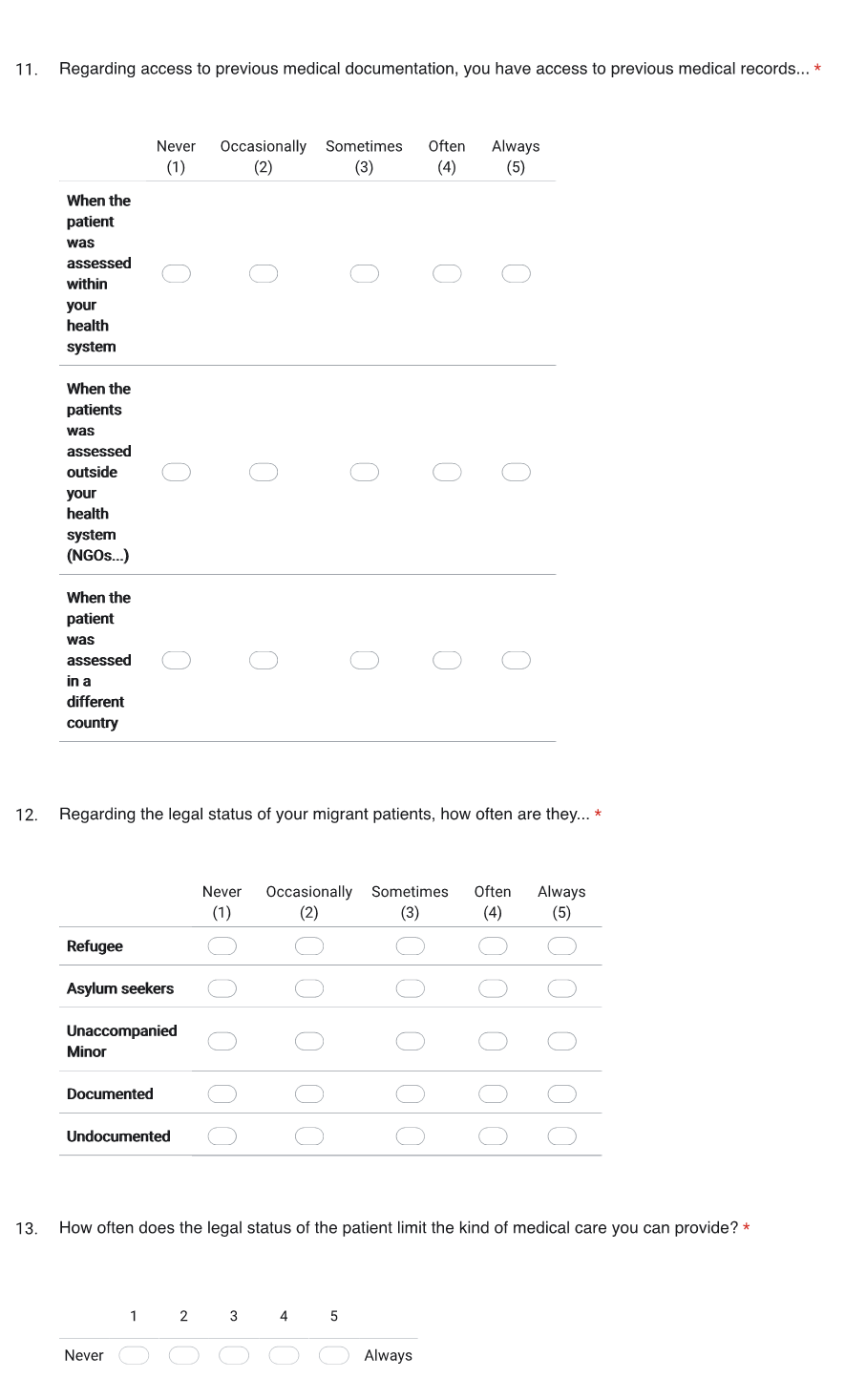
**

**
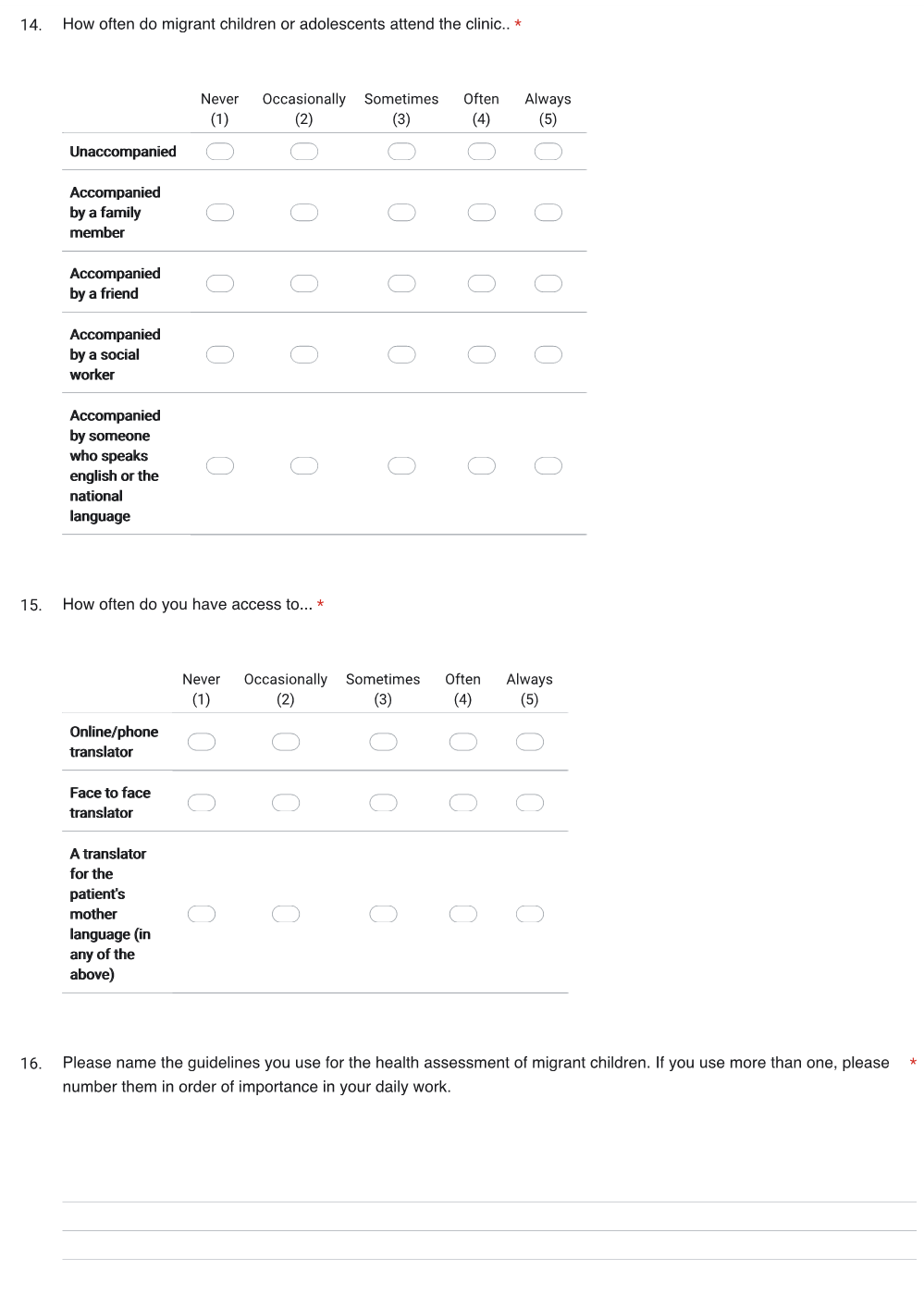
**

**
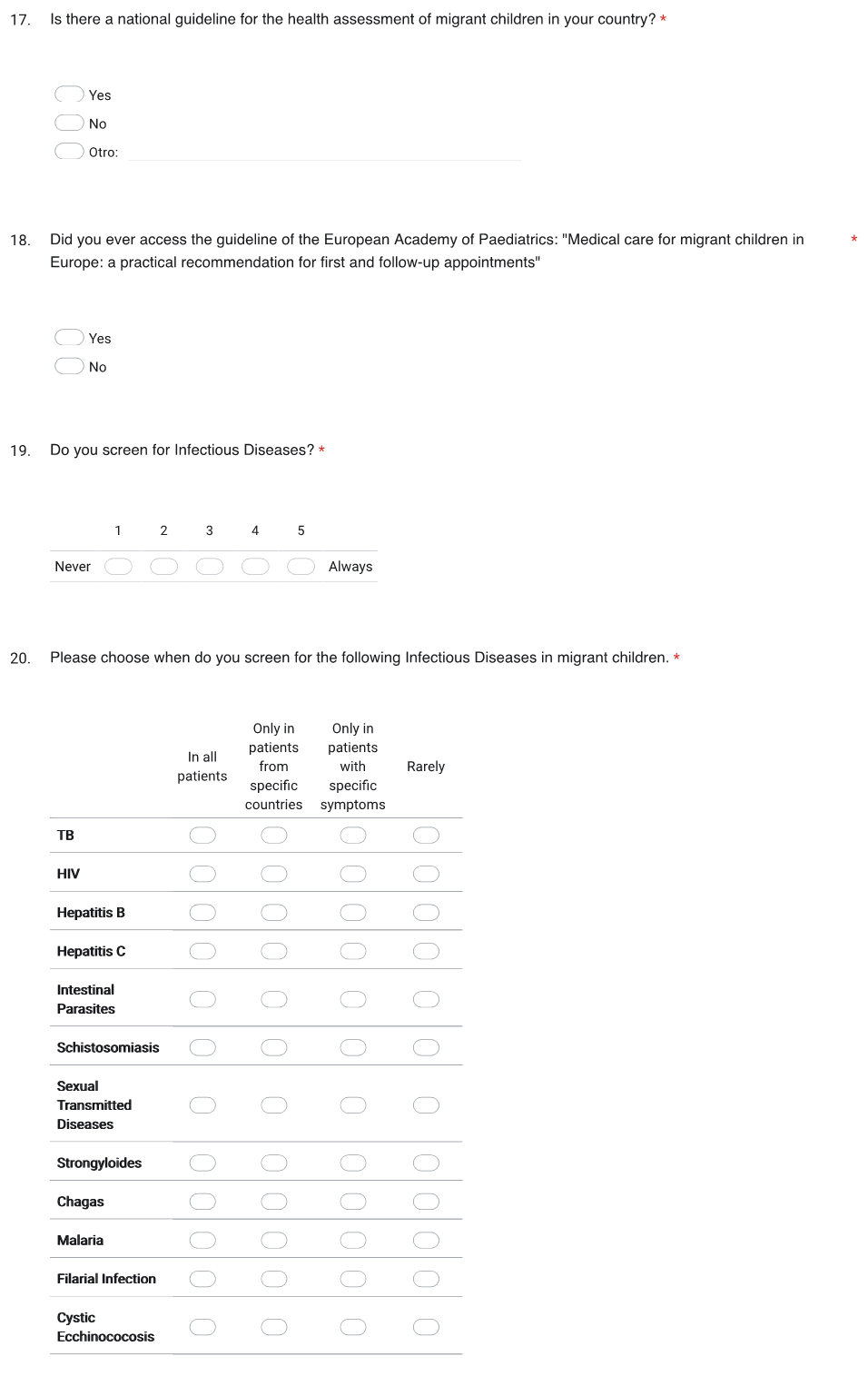
**

**
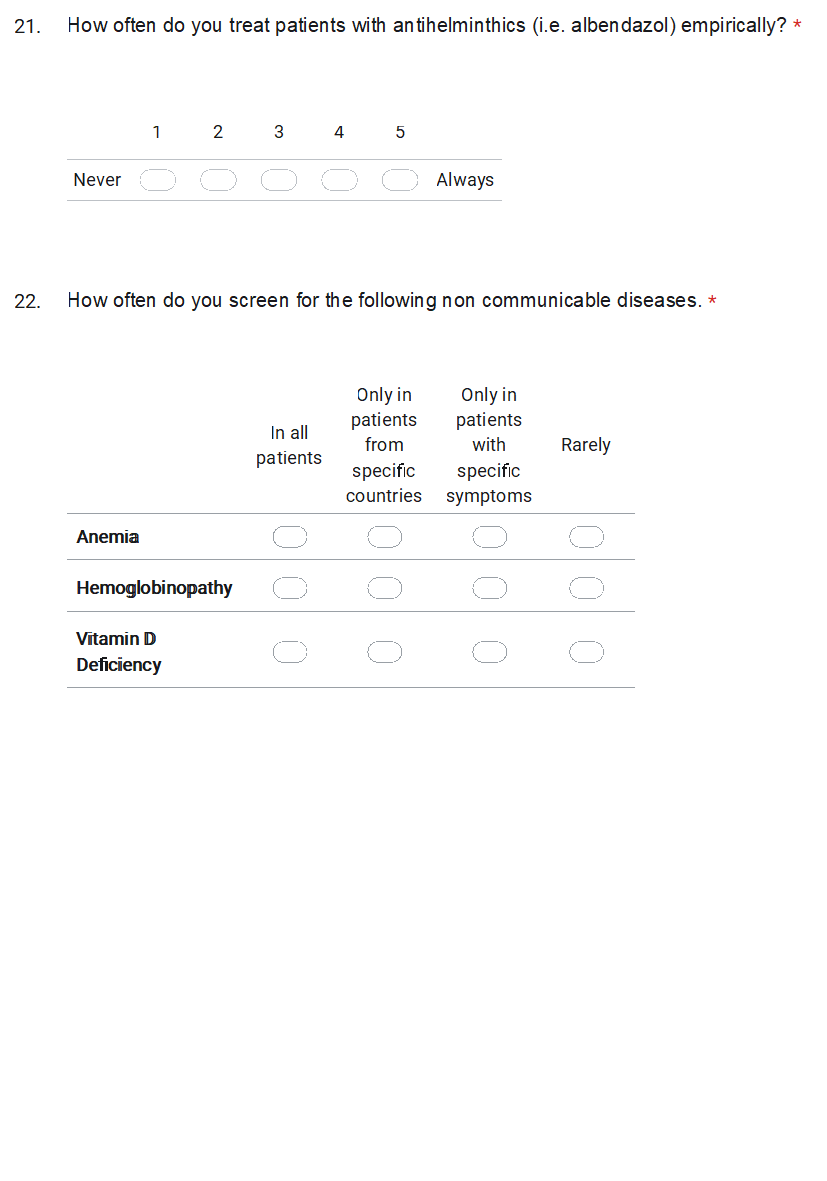
**

**Appendix 2: Topic Guide**

1. **Features of the Initial Health Assessment** - **Comment and Summarise the results of the questionnaire**

To start we will go through some of your questionnaire answers so you can comment on why you do things in a certain way and share challenges and positive ressources you find at each point.

| **Key Areas** | **Headline Questions?** | **Themes** | **Subtopic** | **Potential questions** |
| --- | --- | --- | --- | --- |
| ID Screening | *Comment on Questionnaire answers.* | TB |  | *Are there specific regulations, perceptions or other barriers that stop you doing these* |
|  |  | HIV |  |  |
|  |  |  |  |  |
|  |  | Hepatitis |  |  |
|  |  |  |  |  |
|  |  | Intestinal Parasites | Empirical Treatment with Albendazole? |  |
|  |  | Schistosomiasis |  |  |
|  |  |  |  |  |
|  |  | STD |  |  |
|  |  | Other | Chagas diseases | *Are there things you feel are more important etc?* |
|  |  |  | Filarial infection |  |
|  |  |  | Malaria |  |
|  |  |  | Cystic echino. |  |
|  |  |  | Strongyloidis |  |
| Immunisation | *“How do you approach immunisation?”* | Access to previous immunisation | Documentation | How do you identify if there was previous vaccination? |
|  |  | Vaccination | Completion of the entire national vaccination program? |  |
|  |  |  | Only specific vaccines?  - Which vaccines? |  |
|  |  |  | - Where does vaccination occur? |  |
|  |  |  | SOP for catch-up/ accelerated vaccination? |  |
| Non-communicable diseases + Developement  (Non-ID Screening)  **Paediatric population. | *In younger children, how do you approach growth and development?* | Growth and Nutrition | - parents/carers have concerns about their child’s growth/nutrition |  |
|  |  |  | diet, nutrition and access to healthy food |  |
|  |  |  | centiles for body mass index |  |
|  |  |  | vit D and iron deficiency anaemia | *here check questionnaire answers* |
|  |  | Development and Education | -parents/carers have any concerns about their child’s development? |  |
|  |  |  | - developmental or learning difficulties? |  |
|  |  |  | Vision and Hearing loss |  |
|  |  |  | previous and current access to education, and whether extra support was needed? |  |
|  |  | Congenital Anomalies |  | *How often?* |
| Sexual Health | *“Regarding sexual health, how do you approach it?”* | Screening | STD (adolescents) |  |
|  |  |  | Female genital mutilation |  |
| Mental Health | *“Next I want to explore with you the issue of mental Health. How do you assess it?”* | Screening  (of Risk Factor and Symptoms) |  | *Do you ask or screen patients for Mental Health?* |
|  |  | Symptoms | for parents: behavioural concerns | What kind of psychological manifestations do you see? |
|  |  | Medication |  |  |
|  |  | Available expert (psychiatrists and psychologists) | -Referral | Do you have access to a psychologist or psychiatrist? |
| Pathway | *“From a health care perspective, where do patients come from and where do they land afterwards?”* | -previous access to health care | primary care? |  |
|  |  | -posterior access to health care |  |  |
|  |  | - social/living situation  -since when in country |  |  |
|  |  | Follow-up appointment |  | *“How often do they have a follow up ?”*  “In the same or different clinic?” |
| Documentation System | *“What kind of documentation system do you use?*” | Digital or Paper | Access for future doctors |  |
|  |  | Inside the region and  country  Outside the country | Access to previous medical records |  |
|  |  | Barriers | Time consuming?  Lack of prev medical history? |  |
|  |  | Facilitators | New Tools  Access |  |
| Translators | *“How relevant is the language barrier in your work?”* | Access  Quality |  |  |
| Guideline | *“I would like to hear your thoughts on the available guidelines?”* | National Guideline | for ID screening |  |
|  |  |  | for refugee assessment? or Migrant children? |  |
|  |  |  | Access |  |
|  |  |  | Quality | Comprehensive? |
|  |  |  | Adapted to your context? | Designed for Primary Care or Second/Tertiary Care? |
|  |  | European Guideline (EPA) | Access  Quality  Adapted to your context? |  |
|  |  | Other guidelines? | European… |  |
| Training | *“Are there trainings/courses on migrant health available in your area?”* | availability  access  quality |  |  |
| Financial limitations | *“What role does funding play in your assessment?”* | Barriers | -Funding of Salaries for Health care workers  -Lab and tests | *Comment on questionnaire answer about limitations on care* |
|  |  | Facilitators | -who pays? |  |

2**. Barriers, Facilitators and Suggestions**

Introducing the last part of the discussion:

*“We have now gone through the main aspects of the health assessment, and I would like to discuss with you the Assessment from a more overall perspective. “*

| **Key Areas** | **Headline Questions?** | **Themes** | **Subtopic** | **Potential questions** |
| --- | --- | --- | --- | --- |
| Facilitators/ positive | *“And what resources or other facilitators do you find most helpful?”* | -Improvements in the last years. |  | *How have the guidelines help you offer better care?* |
| Barriers/ negative | *“In your experience, which have been the main challenges in the assessment of these patients so far?”* | -Lack of Resources  -Moving Patients  - Lack of Training |  |  |
| Suggestions for the future | “ *In your opinion, what should be done to improve the health assessment of migrant children in the next years? “* | Evolution in care since last migrant crises? |  | *What new tool have help you provide a better care since you first started working with refuge*es? |

**Appendix 3: Coding Framework**

| THEMES | SUBTHEMES | CODES |
| --- | --- | --- |
| Service structure | Clinical Presentation |  |
|  | Health care Pathway | Posterior Health care |
|  |  | Previous Health care |
|  | Access |  |
|  | Coverage |  |
|  | Funding | Funding available |
|  |  | Funding challenges |
|  | Level of Care | Describing the level of care |
|  |  | Challenges in other levels of care |
|  | Expertise in the service |  |
|  | Type of patients | Accompanied minors |
|  |  | Unaccompanied minors |
|  |  | Undocumented Patients |
| Features of the Health Assessment | History taking |  |
|  | Physical Examination |  |
|  | Immunisation | Access to immunisation status |
|  |  | Serology Screening |
|  |  | Place of vaccination |
|  |  | Documentation of the vaccines |
|  |  | Vaccination Guidelines |
|  | Infectious Diseases | Infectious Diseases Screening |
|  |  | Empirical Treatment with Albendazole |
|  |  | Tuberculosis Screening |
|  |  | Other ID mentioned |
|  | Non-Communicable Diseases | Growth and Development |
|  |  | Nutrition |
|  |  | Vision and Hearing |
|  |  | Chronic Diseases |
|  |  | Blood tests |
|  | Sexual Health | Sexually Transmitted Infections |
|  |  | Female Genital Mutilation |
|  |  | Sexual Education |
|  |  | Other Sexual health issues |
|  | Mental Health | Mental health Screening tool |
|  |  | Referral to Mental health professionals |
|  |  | Education |
|  |  | MH Symptoms |
|  |  | Other MH issues |
|  |  | Other MH tools |
|  |  | Violence and Abuse |
|  | Assessment lenght |  |
| Ressources in the service | Translation services | Access to Translation Services |
|  |  | Type of Translators |
|  | Documentation | Access to International Documentation |
|  |  | Access to National Documentation |
|  |  | Description of their documentation system |
|  |  | Migrant specific documentation |
|  | Other staff within the service | Social Workers |
|  |  | Other available Non Medical roles |
|  |  | Expressing the need for non medical staff |
|  | Guidelines and Training | Adaptability of the gudilenes |
|  |  | Access to Trainining |
|  |  | International Guidelines |
|  |  | National Guideline |
|  |  | Vaccinations Guideline |
| Other challenges | Lack of institutional communication and burocracy |  |
|  | Living situation of the patients |  |
|  | Time |  |
|  | Emotional burden |  |
|  | Patients Relocation |  |
| Other facilitators | Civil Society |  |
|  | New tools | Networks |
|  |  | Digital tools |
| Suggestions |  |  |

**Appendix 4:**

| THEMES | BARRIERS | FACILITATORS | QUOTATIONS |  |
| --- | --- | --- | --- | --- |
| *1. Service Structure* | | | | |
| Level of Care | - In hospital-based services: far from local community follow-up challenging, sometimes unable to vaccinate. - In primary care-based services: fewer resources such as translators, phlebotomy and laboratory support. | - In hospital-based services: more resources to offer an in-depth health assessment. - In primary care based services: closer to the community, easy access, can follow up children and complete vaccinations. - A network between primary and tertiary care. | Barriers:  *”Primary care paediatricians do not have the resources.“* Facilitators*:*  *”It should happen in primary care, where the children live, because many of the problems are social, and that is always much easier to handle if you know who works in the schools or kindergarten.”* |  |
| Access | - Lack of understanding of health system. - Bureaucracy and language barriers, difficult making appointments. | - Easy to access migrant-focused service. | Barriers: *“Sometimes because of the social barriers, not understanding how the system works, not knowing where to refer because of language barriers, they were getting lost in the system.”* |  |
| Coverage | - Delayed health coverage. | - Free services for any migrant. | Facilitators:  *“They are here for their health. And no matter what they are here for, we take them.”* |  |
| Funding | - Lack of staff, space and time to deliver the service due to insufficient funding. - Lack of data to advocate for funding. | - Population specific - funding (eg resettlement schemes). - Network for data collection on refugee health. | Barriers:  *“For these children, I work for free, and that is okay, as long as it is a few children, but, of course, if you do that all day long, then it gets difficult.”* |  |
| *2. Features of the Health Assessment* | | |  | |
| Immunisations | - Limited access to vaccination history, particularly among CYPSAR-U. - Challenges in serological confirmation. | - Available vaccination schedule of country of origin. - Empirical vaccination when vaccination history is unknown based on available guidelines. | Barriers/ Facilitators:  *"Migrant paediatric patients who come with their families. Most often, they bring a vaccination card. In most cases unaccompanied minors never bring a vaccination card."* |  |
| Infectious  Diseases | - Lack of institutional permission infrastructure and staff to perform the infection screening. - Lack of communication with independent TB screening programs. - Logistical barriers around stool sampling and laboratory expertise for parasitological stool analysis. |  | Barriers*:*  *"A separate organization performs the tuberculosis screening, and I am unaware of the results most of the time."*  *"It is very political. As paediatricians, we want that, but, of course, it is about money, but it is also the political and … we could not get the hands together, you know."*  *"We give albendazole independently of stool results. Before, we tried to wait to have the stools, but then I did a study on that and saw that it is almost impossible to have the stool."* |  |
| Non-  Communicable  Diseases | - Language barrier in developmental assessment. - Patient relocation without notification despite serious chronic disease. - High patient relocation due to repeated moves between regions or countries. hinders follow-up. |  | Barriers:  *“We have lost follow-ups for a few children with severe problems like congenital heart diseases, and we have spent a lot of time locating them in camps.”* |  |
| Sexual Health | - Lack of time, privacy and trust, cultural barriers and stigma to discuss sexual health and history of abuse. | - Access to sexual health clinics for further advice. - Possibility to refer patients to gynaecology services with FGM expertise. | Barriers:  *"If there has been a history of abuse, trauma, or rape. then we screen, and sometimes if someone tells us that they have been in prison (…) even if they do not tell you that they have been abused, well...you assume it."*  Facilitators*:  “We have an excellent local sexual health clinic, and they are tuned into working with migrant people so we can refer them there.”* |  |
| Mental Health | - Lack of access to mental health professionals with expertise in migrant health with access to translators. - Lack of training on trauma-based mental health assessment. | - Civil Society association offering mental health support with knowledge of the migrant context. - Access to Psychologists or trained nurses to assess the need for further therapy. - Early enrolment at School. - Appropriate mental health follow-up for proper mental health assessment. | Barriers*: "Our child mental health services are atrocious, I would say; for migrants (they) are almost non-existent.”*  Facilitators*: "One of the key things I found is getting children into education as they get into schools (…), if they integrate, then you know their mental health stabilises.”*  *"There is often a bit of what I call the honeymoon period, (…), but if we see them six months down the line, all the trauma and the problems, you know, they do not go away.”* |  |
| Assessment duration | - Time-consuming assessments, mainly due to extensive history taking. | - Use of Questionnaires for history taking. - Nurse-led history taking. | Barriers: *”Our first visits are 60 to 90 minutes, and then we see them a second time to discuss their results, which is impossible in primary care. ”* |  |
| *3. Resources in the service* | | |  | |
| Documentation | - Absence of access to reliable medical records across migration stages (origin, transit, host) | - Migrant-specific health booklet. | Barriers:  *"Sometimes it is difficult to understand why the child was hospitalised in Afghanistan five years ago."* |  |
| Translation | - Variation on access to translation services within the health care system. - Face-to-face limited languages available. - Telematic: waiting times. - Use of non-official translators (Google translate, friends, workers). | - Face to face: role of translators in conveying cultural contexts. - Telematic: wide range of languages available and more confidential. | Facilitators:  *“I learned many people prefer telephone interpreters. Because they feel it is more anonymous, they can open up better without seeing the person interpreting."*  *"Interpreters are familiar with the patient's culture, and if you ask an inappropriate question, they modify it to get a good answer."* |  |
| Staff | - Lack of non-medical staff within services. Many reported conducting assessments with only a doctor and an assistant, making it challenging to address the broad and complex needs of these patients. | - Community Health Workers: dual health and cultural/linguistic roles. - Case coordinators and social workers - Nurses trained to independently carry out stages of the health assessment. |  |  |
| Guidelines and Training | - Guidelines: not always context-adapted - Information Access: limited data on country-of- origin disease prevalence. - Training: restricted access to migrant health specific programs. | - Guidelines: essential for advocating for resources. National guidelines wer generally avaialble. - Expert Backgrounds: ID and Tropical Medicine expertise highly valued. | Facilitators: *“For us, [the guideline] is very important; otherwise, we can never make our case with the government. ”* |  |
| Teamwork and expert networks | - Bureaucracy and lack of communication between health and other sectors. - Pressure and emotional burden for clinicians. | - Experts network, Scientific Societies - Teamwork: provides holistic solutions for multifaceted care challenges and vital mutual support for clinicians navigating emotional demands. | Barriers: *”Sometimes I feel overwhelmed by the number of things we try to accomplish in one day. I can tell that there are days that I have been feeling burned out (…). This is one of the most difficult things I do as a doctor. ”* |  |
| *4 Other* | | |  | |
| Living condition | - Living situation of patients: lack of access to housing and education. Poor living conditions. | - Civil Society supporting migrants | Barriers:  *“Right now, I have a child hospitalised, a two months old infant, and the parents live on the streets, so they stay hospitalised because she is two months old, and I just cannot discharge her. But everything is full. So recently, I had to release a child, I think, one year old, in the street.”* |  |
